# Surface Modification of 3D Printed Microfluidic Devices for Controlled Wetting in Two-Phase Flow

**DOI:** 10.3390/mi14010006

**Published:** 2022-12-20

**Authors:** Chandler A. Warr, Nicole G. Crawford, Gregory P. Nordin, William G. Pitt

**Affiliations:** 1 Department of Chemical Engineering, Brigham Young University, Provo, UT 84602, USA; 2 Department of Electrical and Computer Engineering, Brigham Young University, Provo, UT 84602, USA

**Keywords:** 3D printing, microfluidic device, droplet formation, surface energy, post-polymerization processing, wetting

## Abstract

Microfluidic devices (MFDs) printed in 3-D geometry using digital light projection to polymerize monomers often have surfaces that are not as hydrophobic as MFDs made from polydimethylsiloxane. Droplet microfluidics in these types of devices are subject to droplet adhesion and aqueous spreading on less hydrophobic MFD surfaces. We have developed a post-processing technique using hydrophobic monomers that renders the surfaces of these devices much more hydrophobic. The technique is fast and easy, and involves flowing monomer without initiator into the channels and then exposing the entire device to UV light that generates radicals from the initiator molecules remaining in the original 3-D polymerization. After treatment the channels can be cleared and the surface is more hydrophobic, as evidenced by higher contact angles with aqueous droplets. We hypothesize that radicals generated near the previously printed surfaces initiate polymerization of the hydrophobic monomers on the surfaces without bulk polymerization extending into the channels. The most hydrophobic surfaces were produced by treatment with an alkyl acrylate and a fluorinated acrylate. This technique could be used for surface treatment with other types of monomers to impart unique characteristics to channels in MFDs.

## 1. Introduction

Microfluidic devices have been rapidly gaining a foothold in advanced technology applications, including biomedical applications such as diagnostic analysis and personalized medicine [1,2]. Several manufacturing methods have been developed for microfluidic devices (MFD) [3], which we will define herein as devices with flow channels less than 1 mm in characteristic diameter. Both image projection (DLP) stereolithography and multijet printing methods have been applied to MFD fabrication, with the former demonstrating significantly higher resolution microfluidic features [4,5]. 3D printing microfluidic devices allows for more complex 3D geometries than some traditional manufacturing methods such as injection molding, but scale-up can be an issue [6]. However, with advancements in the field of 3D printing it has been theorized that by printing many devices at a time (~100) the cost and scalability of this manufacturing method may be commercially viable down the road [7]. While our interest is in medical devices, the same principles of manufacturing and of application apply to MFDs in other technology sectors [8].

One of our main areas of research focuses on forming aqueous droplets in a continuous oil phase [9]. Such droplets have been used as microreactors of pL to nL size in which to interrogate single cells (or particles) in a fluid environment to isolate the cell from other cells and from the external environment. These individualized microreactors have been used for genetic and proteomic analysis of cells [10,11] and of general cell growth when challenged with chemical or biological factors (antimicrobial agents) [12,13]. Often analytical output is conveyed by reporter molecules or reporter reactions from within the droplet. Because these microreactors are used for single cells analysis, it is essential to preclude merging with other droplets. It is also important to eliminate sticking of the droplets to the walls of the channels so the droplets can be conveyed through the MDF to an analytical station. Most often, anti-merging is accomplished by adding surfactants to the oil or aqueous phase, and sometimes by electrostatic charge on the droplets.

In two-phase flow with dispersed aqueous droplets, the oil phase generally consists of fluorocarbons because its high oxygen solubility provides adequate oxygen over time to growing cells [14,15]; however, sometimes hydrocarbon oils can be employed. For droplets in an oil phase, sticking to walls has not been reported to be a large problem, most likely because most MFDs are made by templating silicone rubber in molds etched in silicon [16]. These silicone rubber casts (such as polydimethylsiloxane, PDMS) are bonded to a transparent glass slide or plate using RF plasma treatment [17,18,19], forming an adhesive bond that is sufficiently tight and leak-proof. The silicone rubber walls and floor of the MFD are very hydrophobic, but the upper surface is hydrophilic glass, which is often treated with a commercial surfactant like Rain-X (ITW Global Brands, Houston, TX, USA) to render it fairly hydrophobic [19,20]. When passing 2-phase droplet suspensions, the silicone rubber and hydrophobized glass are sufficiently hydrophobic that aqueous droplets do not adhere in the presence of hydrocarbon or fluorocarbon oils. Wetting by water is precluded because contact of an aqueous fluid with a hydrophobic surface by displacing a hydrophobic fluid is not energetically favorable and does not occur spontaneously [21]. Thus wetting in 2-phase flow in 2-D silicone rubber MFDs has not been an issue for most aqueous droplets in oil.

Our lab has developed and promoted a novel 3-D printing process using acrylate monomers that produces a surface that is less hydrophobic than MFDs made from silicone rubber. This process was developed using a tetrafunctional PEG-diacrylate monomer to give sufficient flexibility (compliant) to the resulting polymer structures that thin film membranes can remain structurally intact during repeated flexing. The PEG diacrylate monomer used in this printing is amphiphilic. PEG is inherently hydrophilic, and the polymer acrylate sections of the polymerized network are much less hydrophilic. Thus the overall polymer chain structure is amphiphilic and is sufficiently dynamic that when confronted with a hydrocarbon based liquid at an interface, the PEG chains can be buried under the acrylate linkages to present a less hydrophilic surface to hydrocarbon surface. We suspect that similar dynamic rearrangement will bury PEG chains when presented with a fluorocarbon or hydrocarbon liquid at the surface. On the other hand, when presented with an aqueous phase at the interface, the PEG quickly migrates to the surface and creates a hydrophilic interface which interacts favorably with the aqueous phase. In the lab, such phenomenon produces what is called “contact angle hysteresis”, in which the surface changes character over time to act more favorably with the liquid it is currently in contact with [22,23]. In a polymeric MFD this phenomenon is manifest by a channel wall being stably wetted by an oil phase (hydrocarbon or fluorocarbon oil) driven by the dynamic burial of hydrophilic groups (like PEG) under hydrophobic polymer groups; in such a case a very brief contact with aqueous droplets will not give time for molecular rearrangement to promote sticking to the wall. However, a prolonged contact (perhaps even shorter than a second) results in dynamic polymer rearrangement and attachment of an aqueous droplet to the surface, and perhaps subsequent spreading of that droplet over seconds or minutes [24].

Our previous publication on MFD design explored forming droplets in our unique annular channel-in-channel (ACC) microfluidic devices printed from various acrylic monomers. In properly designed droplet generators and flow channels, the sticking of droplets to channel walls could be avoided by using more hydrophobic acrylic monomers or by better mechanical design of the droplet generator. However, our previous devices used continual flow (fairly constant flow rate) of at least the continuous phase in order to continually pull off the droplet (dispersed phase) from the orifice of the ACC droplet generator [9]. In that type of flow, adjusting the base monomer was sufficient to form excellent droplets.

**Figure 1:**
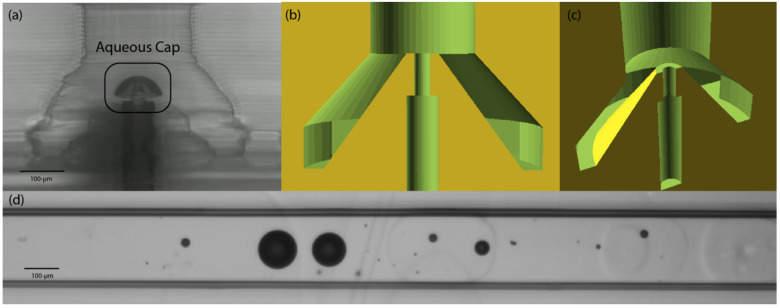
Droplet-on-demand generator printed from PEG-diacrylate monomer. (**a**) Microphotograph of the pedestal of the droplet generator showing the darker aqueous phase spreading over the top of the cone-shaped pedestal. Oil flows up from the outside of the cone, and the aqueous phase flows up the channel in the central core of the cone. (**b**) Schematic showing a side view of the polymerized polymer (in yellow) and the open channels in green forming the cone and pedestal. (**c**) Schematic showing an upward-looking view of the pedestal, which is the light green disk at the top of the darker-green cone. (**d**) Example of droplets and satellites formed when the upper surface of the pedestal is wetted with the aqueous phase.

One of our current efforts is the development of a digital droplet-on-demand (d-DoD) system in which the flow of the aqueous droplets and the continuous oil phases are intermittent and are driven by digital positive displacement pumps built into the microfluidic device [5,7,9,25,26,27], as opposed to constant pressure flows or forced flows from syringe pumps. During these start and stop flows, a droplet may contact a wall for more than a few hundred milliseconds. Even more problematic is that during formation of aqueous droplets with digital pumping systems, the aqueous droplet is perched on or in the orifice of the droplet generator. This leads to wetting and spreading of the aqueous phase at the aqueous-oil interface at the orifice of the droplet generator. The pulsatile nature of our d-DoD system produces a pause in time during droplet formation in which the aqueous phase rests at the water/oil junction at the top of the droplet generator (called the pedestal). During each pause of several hundred milliseconds, it appears that the aqueous phase slightly but progressively spreads along the amphiphilic polymer material and progressively increases the wetted area at the top of the pedestal. As this wetted area increases over time the droplet formation arises from a wider base, resulting in satellite droplets forming in the d-DoD system. As seen in Figure 1a, the wetted region on top of the pedestal is much larger than the designed area intended for the release of fluid for droplet formation (Figure 1b). These droplets are often accompanied by more satellite droplets (see Figure 1d) whose number increases over time as the top of the pedestal is progressively wetted by the spreading aqueous phase. Ideally, all particles or cells intended to be encapsulated should be captured within a main droplet, not inside satellite droplets. Production of satellite droplets also decreases the volume of the primary drop. We hypothesized that increasing the hydrophobicity of the 3D-printed PEGDA material would decrease the magnitude (both number and size) of these satellite droplets.

Initial experimentation to coat the internal microfluidic channels of the 3D-printed PEGDA polymer showed that it is possible to coat the channels using an acrylate monomer with a hydrophobic group attached to it. The current processing protocol for our 3D-printed devices involves a post-exposure which increases the degree of polymerization in the printed material, thus increasing the lifespan of delicate features. This post-exposure is done using a 430 nm light source, which is outside of the effective range of the UV absorber, but still within the range of the photoinitiator. Thus additional polymerization can occur after the initial printing and removal from the printer. The fact that this increases the lifespan of these features and the hardness of the material means that there must be viable photoinitiator and perhaps monomer still present in the polymer [7,25]. It was postulated that some unreacted acrylate groups were also present on the surface of the channel walls and that those may still be available to react with additional monomer. We theorized that this viable photoinitiator and possible unreacted monomers could be used to locally react with a “post-printing-added” acrylate monomer on the surface of the channel walls. Our results show that this is the case and that more hydrophobic surfaces can be attained.

Other published approaches to controlling material and surface properties for parts made by stereolithographic printing include using more than one material in the same print [28,29]. While potentially possible for producing hydrophobic surfaces on a channel by using a hydrophobic print material, this greatly slows down the printing while one material is being swapped out for another material in the printer. For multiple channels with complex geometries, such a process may not be feasible because formation of a vertical channel would require manually swapping monomers during every layer of print.

## 2. Materials and Methods

### 2.1. Custom 3D Printer

The 3D printer used in this study was custom made and is described in more detail in other publications [5,7,25,27,30,31]. Briefly, the printer is a digital light projection (DLP) style 3D printer using a Visitech 2560 × 1600 pixel light engine with a 365 nm LED as the projection source and a 100 mm linear Griffin Motion stage as the *z*-axis control. Additionally, it has several other tip/tilt and focus calibrations built in to bring the custom resin tray in planar focus with the light engine. Custom Python software is used to control calibration parameters and handle printing such that individual printing layers can have their own (or multiple) exposure times depending on the image projected onto the focus plane. This allows for precise control over printing parameters and features.

### 2.2. Materials

**Figure 2:**
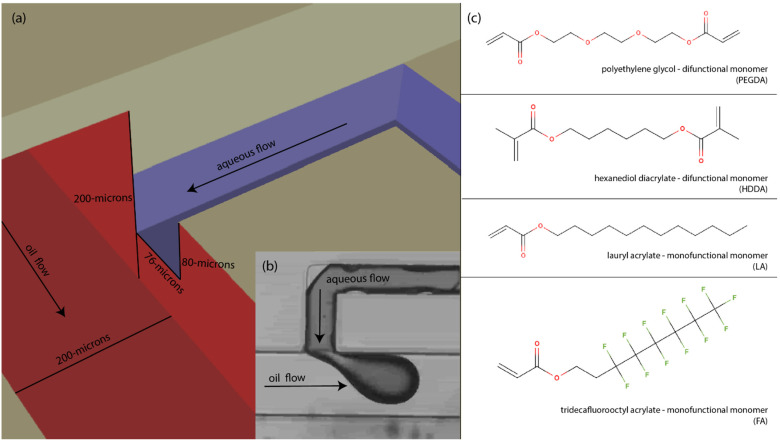
T-junction geometry and monomer chemistry: (**a**) Schematic of cutout cross section of the T-Junction droplet generator with the appropriate dimensions of the oil and aqueous flow channels. (**b**) Micrograph of dyed aqueous fluid forming droplet during operation. In this top-down view the width of the aqueous channel is 76 µm and the width of the oil channel is 200 µm. (**c**) The four photopolymerizable acrylate monomers used in study, including the 3D printing polymer (PEGDA) and the three monomers used for surface treatment (HDDA, LA, FA).

Custom resins were made according to a previously established procedure [30,31] which includes mixing a photopolymerizable acrylate monomer with a UV absorber and photoinitiator. The resin used in this study has been used in several other published studies [5,7,25,27,30,31] and works well to produce consistent features and to print uniformly. It contains a polyethylene glycol diacrylate (PEGDA) monomer, 1% Irgacure 819 as a photoinitiator, and 0.38% avobenzone as a UV absorber. This absorber and photoinitiator combination works well with the 365 nm LED in used in the Visitech light engine. In addition to the 3D printing resin, three other acrylate monomers were used as experimental surface treatments for the study; they are hexanediol diacrylate (HDDA), lauryl acrylate (LA), and tridecafluorooctyl acrylate (FA), which were used as received and whose chemical structures are depicted in Figure 2c. These particular acrylate monomers were chosen because of their hydrophobic chemistry, which potentially could be imparted to the surface of the 3D printed channels. It is worth noting that HDDA is the only difunctional acrylate monomer used in the treatment and has the potential of forming a branched or networked polymer coating. The LA and FA monomers have a single acrylate group with a long hydrophobic tail and are capable only of forming linear polymers attached to the surface.

### 2.3. Optical System

The optical system used to visualize and photograph the MFDs and droplets in this study is a Nikon TE300 inverted microscope equipped with a FLIR Blackfly S USB3 (Teledyne FLIR, Wilsonville, OR, USA) monochrome camera. This camera has high sensitivity and allows computer control of exposure and gain for consistent and comparable image capture.

### 2.4. 3D Printed T-Junction

A central T-junction droplet generator was chosen for this study because it forces contact between the dispersed aqueous phase and the wall during the formation of droplets and is sensitive to any change in surface hydrophobicity. This droplet generator was chosen over other geometries, such as co-flowing (pinch off), because while these other geometries are more practical for MFD design, they are not considered as sensitive for this type of testing in which we encourage (and even force) the aqueous phase to contact the wall. Throughout this study the T-junction droplet generator geometry was held consistent with dimensions as shown in Figure 2a. Briefly, the continuous oil channels have a square cross section with 200-µm height and width, while the aqueous flow channel has an almost square cross section with a 76-µm width and 80-µm height. These dimensions utilize the 7.6 pixel pitch of the light engine as well as the standard 10-µm layer height used in this custom printer. As seen in Figure 2a, the water outlet has smaller channel dimensions than the continuous oil channel and is centered on the face of the wall. This was done in order to avoid contact of the forming droplet with the top (ceiling) and bottom (floor) surfaces but force direct contact of the forming droplet with the adjacent sidewall of the oil channel. Also of note, the dispersed aqueous phase was dyed to aid in visual differentiation between the two immiscible phases.

### 2.5. Acrylate Monomer Treatment and Optical Exposure

The acrylate monomer postprint treatment was performed according to the following procedure. (1) The device was removed from the printer and thoroughly washed with isopropyl alcohol (IPA) including all internal microfluidic channels. (2) The hydrophobic monomer was then injected through the relevant fluid channels by hand with a small syringe. (3) The device was then immediately placed under the postexposure station for the UV treatment. The post exposure station consists of a UV postexposure using a 430 nm LED within a 3D printed housing. As mentioned above, this wavelength LED was chosen because it falls within the excitation range of the photoinitiator while being outside of the effective range of the UV absorber, which means that additional polymerization could occur within the polymer (or on the polymer surface) after the initial print is complete. A postprint exposure like this is very common among commercial printers using this same polymerization method. In the experimental design, the treatment dose was varied by changing the exposure time each device had within the post curing station. The maximum treatment dose was initially determined to be a dose sufficient to form walls around a channel, but not cause polymerization within a channel during the normal MFD printing protocol. For example, an optical dose in which during normal printing caused the monomer to not be cleared (by flushing with isopropanol) from the channel, was decreased by 20%; this optical dose was tested again (and decreased again if needed) to iteratively find a dose that would repeatedly clear, and above which the channel would not clear of the monomer. This was the UV dose (time and intensity) used subsequently during postprint treatment with candidate monomers in the channels. In some experiments, multiple treatments were executed sequentially. Since the monomers used in postprint surface treatment did not contain photoinitiator, we did not expect polymerization to occur in the lumen of the channels, but only upon the surface where residual photoinitiator from the original 3D print might reside.

## 3. Results and Discussion

We hypothesized that it would be possible to use the postulated presence of unreacted initiator at the surface of previously formed channels to polymerize a layer of hydrophobic acrylate monomer. We evaluated this hypothesis by evaluating any change in the surface hydrophobicity by forming water-in-oil droplets in these devices and forcing them against the treated surface. This method of using residual initiator and fresh acrylate monomer to polymerize a surface coating could in theory be used for many other applications to impart particular surface chemistry in MFD channels; however in the study we only investigated the surface hydrophobicity. Several different treatment conditions were examined using three hydrophobic monomers, and then the monomer that best enhanced surface hydrophobicity was used on the ACC droplet generator to investigate how digital droplet generation was influenced.

### 3.1. Treatment Conditions Using Acrylate Monomers with Residual Initiator

3D printed microfluidic devices were manufactured containing the previously described T-Junction droplet generator followed by a long serpentine channel, a section of which had a narrow channel to force contact between the droplets and the channel wall. Using the treatment protocol described above, some MFDs were put through several cycles of treatment with each of the monomers in order to increase any effect the hydrophobic monomers may have had on the channels. Evaluation involved rating the effectiveness of various treatments on both droplet formation and static (no flow) droplet contact in the narrow channel.

**Figure 3:**
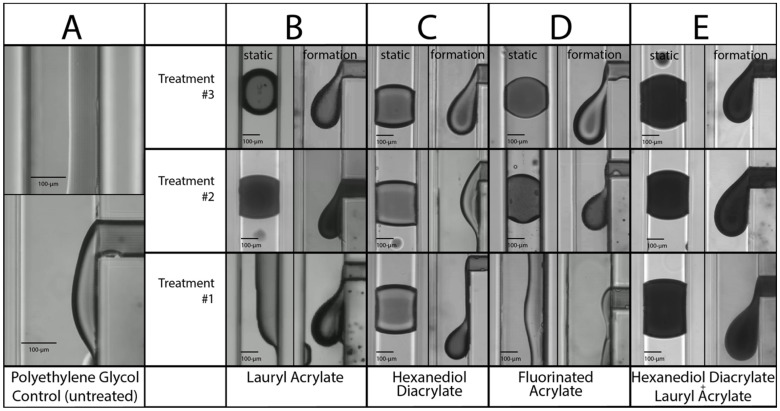
Droplet generation from T-junction with various hydrophobic acrylate coatings; each treatment involved a coating of the acrylate monomer followed by a UV exposure and washing with IPA. Multiple treatments were used to increase the effectiveness of the coatings. Column (**A**): PEG-diacrylate-polymerized T-junction (lower) and serpentine channel (upper) showing 2-phase flow and no droplets formation on untreated polymer. (**B**): Droplet formation on base-polymer treated once (lower), twice (middle) or three times (upper) with lauryl acrylate. (**C**): Droplet formation on base-polymer treated once (lower), twice (middle) or three times (upper) with hexanediol diacrylate. (**D**): Droplet formation on base-polymer treated once (lower), twice (middle) or three times (upper) with fluorinated acrylate. (**E**): Droplet formation on base-polymer treated once with hexanediol diacrylate and then treated once (lower), twice (middle) or three times (upper) with lauryl acrylate.

The results are shown in Figure 3, in which the darker phase is the dispersed aqueous phase. Both “formation” and “static” images are shown to reveal how a droplet forms at the T-junction (formation) and how the droplet wets after formation during static (no flow) contact with the channel walls. Micrographs of the flow in the base PEGDA material are shown in column A of Figure 3. No consistent droplet formation was observed in the native PEGDA material, and downstream of the droplet generator a coflowing phase separated flow was seen as previously reported [9]. Initial testing of the hydrophobic monomer treatments showed that there was indeed a difference in the droplet formation ability of the material after a single treatment. Furthermore, we found that multiple cycles of identical treatment enhanced the hydrophobicity, so three serial treatments were performed with the different hydrophobic monomers. As shown in column B of Figure 3, one treatment of lauryl acrylate was insufficient to allow for consistent droplet formation dramatically different from the PEGDA control. However, when serial treatments were applied, the qualitative hydrophobicity was improved with each subsequent treatment; after the third treatment a non-spreading droplet can be seen forming with the T-junction geometry. Columns C and D of Figure 3 show a similar trend for the results of treatments using hexanediol diacrylate and the fluorinated acrylate. All three individual hydrophobic monomers show progressive improvement for subsequent treatments, and after three photo-treatments all monomer types show comparable droplet formation results.

An additional test condition was performed which used a single hexanediol diacrylate base treatment followed by three standard lauryl acrylate treatments (see column E of Figure 3). Rationale for this treatment was based on the postulate that the HDDA monomer may present additional functional groups on the surface after the first treatment because of its two acrylate groups, compared to the other two monofunctional acrylates tested. However, after three treatments of the lauryl acrylate following the HDDA base layer, no significant improvement was observed compared the other monomer treatments.

These results show that the method of attaching hydrophobic acrylates via residual UV initiator at the surface increases the hydrophobic quality of the microfluidic channels such that droplet formation becomes practical, as demonstrated with a variety of monomer treatments. It is also worth noting that there is likely some interaction between the continuous oil phase and the hydrophobic monomer that has an effect on droplet formation. For example, a hydrocarbon mineral oil was used as the continuous phase for these tests, which may be more chemically compatible with the lauryl acrylate compared to the fluorinated acrylate; likewise, a fluorinated oil may wet the channel wall better when coated with the fluorinated acrylate.

We propose that a significant benefit of this technique is that it provides a covalent attachment of the modifying molecules to the surface of channels in the MFD. Other methods that involve surfactact coatings or non-reactive processes may have potential to be removed over time. More research is needed to validate the persistence of the surface coating over hours or days of flow. These results suggest that this technique may be useful to covalently attach other functional groups onto the surface of a DLP 3D-printed microfluidic device—molecules such as proteins for biological anchorage, carbohydrates for cell signaling, fluorescent acrylates for visualization, or other such applications. We note that the results presented herein only explored a small subset of possible surface treatments. In addition to other types of monomers, future work can include more variations in post-exposure time, intensity, and wavelength, and perhaps in the initiator concentration.

### 3.2. Application in Annular Channel-in-Channel Droplet Generator

**Figure 4:**
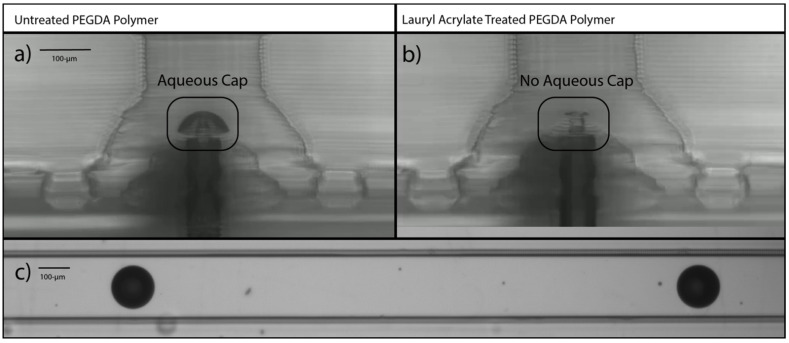
Before and after images of droplet generator. (**a**) Microphotograph of droplet generator made from PEG-diacrylate with no surface treatment after several minutes of droplet generation, showing an aqueous phase in the form of a cap attached to the top of the pedestal. (**b**) Microphotograph of droplet generator made from PEG-diacrylate with three serial treatments of lauryl acrylate, showing no aqueous cap on the pedestal after several minutes of droplet generation. (**c**) Microphotograph of droplets flowing in a serpentine channel, which droplets were formed in the MFD made with three serial treatments of lauryl acrylate.

For subsequent investigations on our ACC droplet generator, the three-fold treatment with lauryl acrylate was used because of the ease of use of the monomer (low viscosity), low cost, and rapid curing treatment times. The three-fold surface treatment with FA was applied to the d-DoD system. Figure 4a shows the droplets formed with the native PEGDA polymer without any additional treatment. Many satellite droplets were seen (see Figure 1d), and the top of the pedestal of the droplet generator had an obvious aqueous ring around it that was hypothesized to be creating spurious satellite droplets during drop formation. We hypothesize that the stretched aqueous phase has a larger cross section that breaks into a primary drop and many satellite droplets, rather than a single primary drop.

Figure 4b show the same d-DoD geometry during droplet formation (there is aqueous phase in the core of the cone) after the three treatments of lauryl acrylate post-processing, which shows no aqueous film attachment at the top of the pedestal and much cleaner droplets formed throughout the production of droplets (Figure 4c), indicating the effectiveness of the lauryl acrylate postprocessing treatment in forming better (consistently reproducible) droplets. For example, please compare Figure 4c to Figure 1d. Future work with high speed cameras could be used to confirm this hypothesized formation process.

## 4. Conclusions

We have successfully investigated and developed a post-printing process that reduces the wetting and adhesion of aqueous droplets in a continuous oil-phase flow in a microfluidic device. The procedure consists of taking a 3D printed microfluidic device made from PEG diacrylate monomers, rinsing the unprinted monomer from the flow channels, refilling the flow channels with a different and more hydrophobic monomer not containing initiator, and then exposing the entire device to UV light to initiate polymerization of the hydrophobic monomer to the channel surfaces. Following exposure the unpolymerized monomer was rinsed from the MFD. Results show that lauryl acrylate, fluorinated acrylate, and hexanediol diacrylate increased the hydrophobicity of the channel surfaces. Repeated surface treatments increased the hydrophobicity further. This same method can have application in applying other surface coatings to MFDs, such as acrylate monomers having functional groups that are fluorescent, that possess unique chemistry or that present charged groups at the surface, that bind or repel targeted chemicals in the flow, or that contain proteins or nucleic acid groups for binding of cells or DNA to the surface. This surface treatment has great potential to provide unique chemistry in MFDs.

## Data Availability

There is no numeric data associated with this study. Photographs can be obtained by contacting the authors: chandlerwarr@gmail.com or pitt@byu.edu.
